# Applicability of Traditional In Vitro Toxicity Tests for Assessing Adverse Effects of Monoclonal Antibodies: A Case Study of Rituximab and Trastuzumab

**DOI:** 10.3390/antib7030030

**Published:** 2018-08-17

**Authors:** Arathi Kizhedath, Simon Wilkinson, Jarka Glassey

**Affiliations:** 1 School of Engineering, Newcastle University, Newcastle Upon Tyne NE17RU, UK; arathikmenon.2004@gmail.com; 2 Institute of Cellular Medicine, Newcastle University, Newcastle Upon Tyne NE2 4HH, UK; simon.wilkinson@newcastle.ac.uk

**Keywords:** biopharmaceutical development, in vitro tests, off target toxicity, developability, traditional testing, monoclonal antibodies

## Abstract

Monoclonal antibody (mAb) therapeutics have a promising outlook within the pharmaceutical industry having made positive strides in both research and development as well as commercialisation, however this development has been hampered by manufacturing failures and attrition. This study explores the applicability of traditional in vitro toxicity tests for detecting any off-target adverse effect elicited by mAbs on specific organ systems using hepatocarcinoma cell line (HepG2) and human dermal fibroblasts neonatal (HDFn), respectively. The mechanism of antibody dependent cytotoxicity (ADCC), complement dependent cytotoxicity (CDC) via complement activation, and complement dependent cellular cytotoxicity (CDCC) were assessed. Major results: no apparent ADCC, CDCC, or CDC mediated decrease in cell viability was measured for HepG2 cells. For HDFn cells, though ADCC or CDCC mediated decreases in cell viability wasn’t detected, a CDC mediated decrease in cell viability was observed. Several considerations have been elucidated for development of in vitro assays better suited to detect off target toxicity of mAbs.

## 1. Introduction

Monoclonal antibody (mAb) therapeutics currently dominate many therapeutic areas such as oncology and rheumatism. The non-clinical safety testing of mAbs however are different and more complex when compared to small molecules owing to innate differences in structure, clearance, mechanism of action, and specificity of immune responses elicited [1]. The main considerations for development of non-clinical safety testing strategies for mAbs are: co-incubation of cell line of interest with immune responsive cells; optimisation of cell density and incubation times; and choice of off-target organ system and assay endpoint. Furthermore, the innate complexity, diversity, and size of mAb based therapeutics intensify the need for carefully designed in vitro systems that accounts for the above factors. 

Effector functions of mAbs such as phagocytosis, antibody dependent cytotoxicity (ADCC), complement dependent cytotoxicity (CDC) via complement activation, and complement dependent cellular cytotoxicity (CDCC) are regulated by the interaction between the Fc region of mAbs with the receptors on immune cells [2,3].This requires the co culture of target cells with immune responsive cells, such as Peripheral Blood Mononuclear Cells (PBMCs), to elicit the immune response pre requisite for causing adverse effects that could lead to off-target toxicity. PBMCs comprise of B cells, T cells, monocytes, dendritic cells, and natural killer cells and these cells express various Fc gamma receptors which bind to the Fc region of IgG mAbs and induce effector functions like ADCC, phagocytosis, transport, and catabolism [4]. For complement activation and CDC, exposure to human serum containing complement proteins is required [5]. A combination of PBMCs and complement protein is used to assess potential CDCC where a primary binding to complement protein is followed by engaging with complement receptors on natural killer cells or macrophages [2].

**Figure 1:**
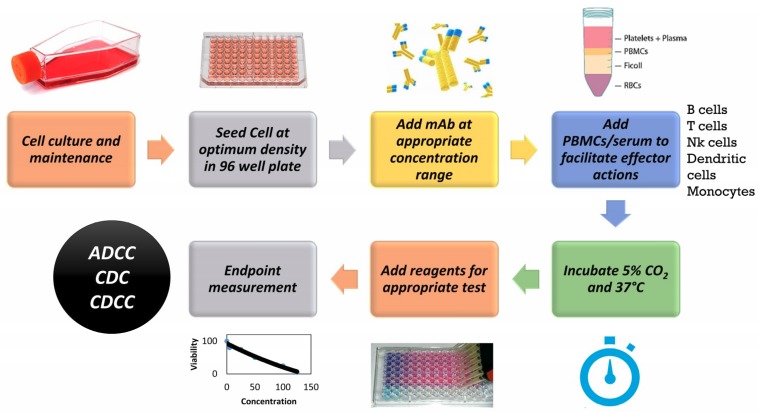
General Methodology for an in vitro assay to detect toxicity of monoclonal antibody (mAb) based therapeutics. PBMCs: Peripheral Blood Mononuclear Cells. ADCC: Antibody dependent cytotoxicity. CDC: Complement dependent cytotoxicity. CDCC: Complement dependent cellular cytotoxicity, NK: Natural Killer, RBCs: Red Blood Cells

Different in vitro toxicity assays are in place for assessing toxicity endpoints and these have been described in detail in multiple studies and reviews [6]. Of these assays, WST-1 assay is routinely used for assessing cytotoxicity of compounds. WST-1 is a tetrazolium salt that is converted by mitochondrial dehydrogenase enzymes into a soluble coloured formazan compound which can be quantified using absorbance endpoint measured using a spectrophotometer. The absorbance values are reflective of mitochondrial enzyme activity which is a measure of the metabolic activity of cells. Another sensitive marker for cell viability is by measuring the adenosine triphosphate (ATP). As the cells lose membrane integrity, they fail to synthesize ATP and any remaining ATP in the cytoplasm is rapidly depleted by ATPases which are enzymes that catalyze the dephosphorylation of ATP into ADP. [7]. CellTiter-Glo^®^ Luminescent Cell Viability Assay (Promega, UK) allows for detection of metabolically active cells through quantification of adenosine triphosphate (ATP). Luciferin upon interaction with ATP emits light in a reaction catalysed by firefly luciferase and this can be measured by recording the luminescence. Potential cytotoxicity and reduction in ATP levels of hepatocarcinoma cell line (HepG2) and human dermal fibroblasts neonatal (HDFn) cells following exposure to mAbs were investigated using the methodology described in Figure 1.

Previous studies indicate that following immunogenicity, hepatotoxicity and dermal toxicity are the two main adverse effect categories associated with mAbs [8]. This report aims to assess the suitability of traditional toxicity assays to investigate potential organ/system related adverse effects of mAbs that could lead to hepatotoxicity and dermal toxicity using hepatocarcinoma cell line (HepG2) and human dermal fibroblasts neonatal (HDFn), respectively. 

The mAbs used in this case study are rituximab and trastuzumab. Rituximab is an AntiCD20 chimeric monoclonal antibody, with an IgG1 heavy chain and kappa light chain, used for therapeutic indications such as Non-Hodgkin’s lymphoma, chronic lymphocytic leukaemia, rheumatoid arthritis, granulomatosis with polyangiitis, and microscopic polyangiitis. It has been shown to elicit hepatobiliary and skin related adverse effects in addition to immunogenicity and hypersensitivity [9]. Trastuzumab is an AntiHER2 humanised monoclonal antibody, with an IgG1 heavy chain and kappa lights chain, indicated for breast cancer and gastric cancer. It has shown to cause infusion related reactions, hypersensitivity as well as hepatobiliary, and skin related adverse effects [10]. The concentrations of mAbs chosen were based on their relevance to in vitro testing perspective as they will be lower than the recommended dosing in clinic. The recommended dosing for trastuzumab and rituximab vary for different forms of administration as well as therapeutic conditions. For rituximab cells containing CD20 surface markers the half maximal inhibitory concentration (IC50) is around 0.2 μg/mL [11] and for trastuzumab it is 24 μg/mL for gastric carcinoma cell lines [12]. The concentrations chosen in this study are around these ranges to assess the off target toxicity and have been used in previous studies that assessed CDC, CDCC, and ADCC effector mechanisms of mAbs in in vitro studies [13].

## 2. Materials and Methods

### 2.1. Materials and Reagents

Freshly isolated peripheral blood mononuclear cells (PBMC), human universal AB serum, rituximab (stock 100 mg/mL) and trastuzumab (600 mg/mL) were kindly provided by Alcyomics Ltd., Newcastle upon Tyne, UK. Dulbecco’s Modified Eagle Medium (DMEM, high glucose, with bicarbonates), Fetal Bovine Serum (FBS), Pencillin-Streptomycin (10,000 units penicillin and 10 mg streptomycin/mL), Phosphate Buffered Saline (PBS), Trypsin Ethylenediaminetetraacetic acid (EDTA) solution, Minimum Essential Medium (MEM) non-essential Amino acids, l-glutamine solutions 200 mM and Sodium Pyruvate Solution, tissue culture flask, 96 well F-bottom plates and WST-1 (Cat. No: 05015944001) were purchased from Sigma Aldrich, Dorset, UK. CellTiter-Glo^®^ Luminescent Cell Viability Assay was purchased from Promega, Southampton, UK. All kits will be used as per manufacturers’ instructions.

### 2.2. Cell Culture and Maintenance

HepG2 (ATCC^®^ HB-8065™) and HDFn (ATCC^®^ PCS-201-010™^)^ cells, kindly provided by the Newcastle University BioBank, Newcastle Upon Tyne, UK. were grown as an adherent culture in complete growth media (Dulbecco’s Modified Eagle’s Media supplemented with 10% Fetal Bovine Serum, 1% Penicillin/Streptomycin, 1% Non-Essential Amino Acids, 1% l-Glutamic acid and 1% Sodium Pyruvate) in T75 tissue culture flasks at 37 °C with 5% CO_2_, all of the consumables were purchased from Sigma, Dorset, UK. The cells were subcultured 3 times a week using the following procedure: The spent medium was removed and the cells were given a Phosphate Buffered Saline wash following which 1× diluted Trypsin was added to gently lift the cells. The cells were then re-suspended in 1:15 dilution in T75 tissue culture flasks. 

### 2.3. Cell Seeding and Exposure to mAbs

The HepG2 and HDFn cells were seeded at a density of 5000 cells/well onto a Greiner 96-well F bottom tissue culture plate (Sigma, Dorset, UK). 50 µL of rituximab (R) and trastuzumab (H) were added to the test wells at final concentration of 0.1, 1 and 10 μg/mL upon which the plates were incubated for a further 3 h at 37 °C with 5% CO_2_. Details of the final mAb concentration and volumes associated are shown in Table A1. Appropriate volumes of media were added to the control wells to compensate for volume differences arising due to addition of mAbs and effector cells/serum. A final concentration of 10 μg/mL of human IgG was used as isotype control. 5% (*v*/*v*) of ethanol was used as the positive. Target control refers to the target cells (HDFn or HepG2) in media.

### 2.4. Complement Dependent Cytotoxicity (CDC)

**Figure A1:**
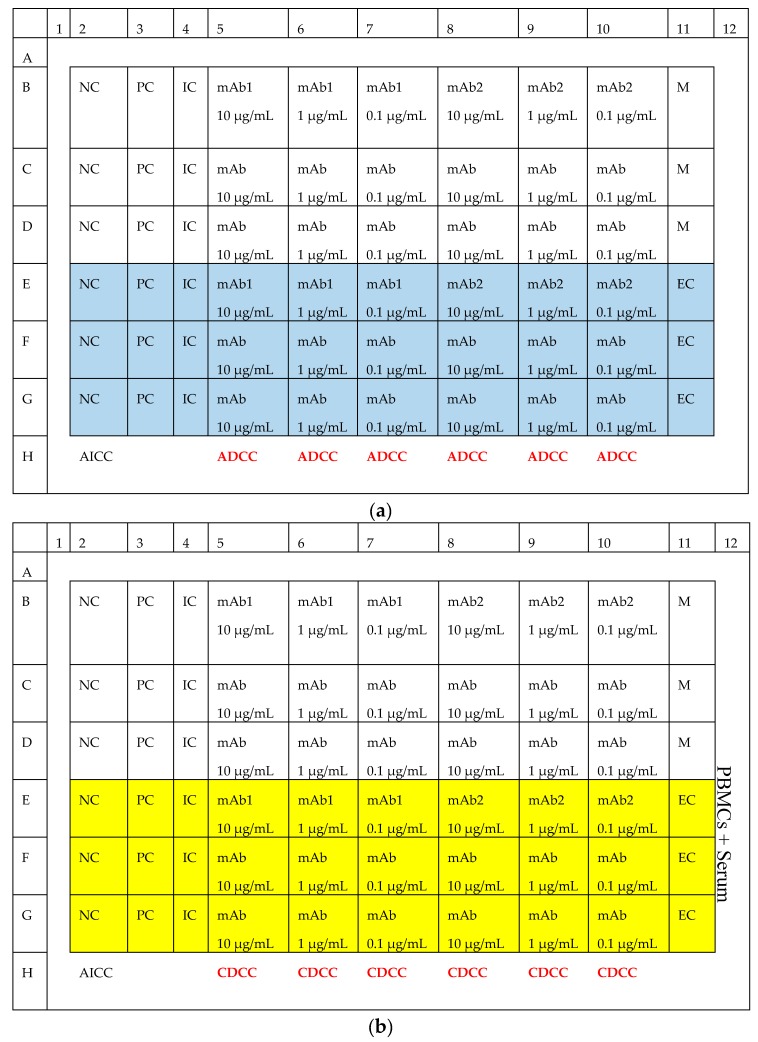

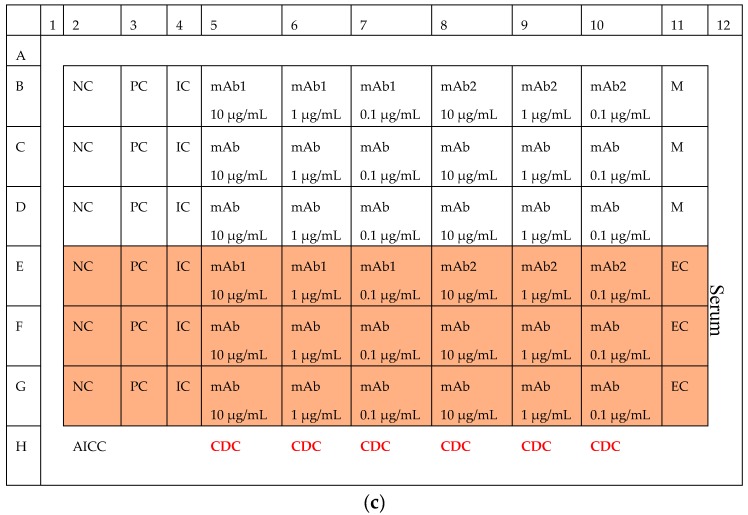
Layout of (**a**) ADCC, (**b**) CDCC and (**c**) CDC experiment. NC: Negative Control, PC: Positive Control, IC: Isotype Control, AICC: Antibody independent cellular cytotoxicity, EC: Effector Control.

Following incubation with varying concentrations of rituximab and trastuzumab, 50 μL of human universal AB serum were added to the test wells. The plate was incubated overnight at 37 °C with 5% CO_2_ [14,15]. The layout of the 96-well plate for CDC experiment is outlined in Figure A1c (Appendix A). 

### 2.5. Antibody Dependent Cellular Cytotoxicity (ADCC)

Following incubation with varying concentrations of rituximab and trastuzumab, 50 μL of PBMCs at a density of 50,000 cells per well were added to the test wells to achieve an effector to target ratio of 10:1. The plate was incubated overnight at 37 °C with 5% CO_2_ [14,15]. The layout of the 96-well plate for ADCC experiment is outlined in Figure A1a. 

### 2.6. Complement Dependent Cellular Cytotoxicity (CDCC)

Following incubation with varying concentrations of rituximab and trastuzumab, 50 μL of human universal AB serum and 50 μL of PBMCs at a density of 50,000 cells per well were added to the test wells to achieve an effector to target ratio of 10:1. The plate was incubated overnight at 37 °C with 5% CO_2_ [14,15]. The layout of the 96-well plate for ADCC experiment is outlined in Figure A1b. 

### 2.7. WST-1 Cell Proliferation Assay

Following exposure of cells to the test compounds for 24 h, 10 μL of WST-1 reagent were added per well and the plates were incubated for an additional 4 h at 37 °C with 5% CO_2_. Endpoint measurements of absorbance were taken at 480 nm and 600 nm (background) on FLUOstar® Omega multimode microplate reader (BMG Labtech, Cary, NC, USA). Cell viability was expressed as a percentage of the target control. 

### 2.8. CellTiter-Glo^®^ Luminescent Cell Viability Assay

Following exposure of cells to test compounds for 24 h, the plate and its contents were equilibrated at room temperature for approximately 30 min. Volume of CellTiter-Glo^®^ Reagent equal to the volume of cell culture medium present in each well (e.g., 100 µL of reagent to 100 µL of medium containing cells for a 96-well plate) was added. Contents were mixed for 2 min on an orbital shaker (VWR microplate shaker, St Neots, UK) to induce cell lysis. The plate was allowed to incubate at room temperature for 10 min to stabilize luminescent signal. Luminescence was recorded in FLUOStar Omega multiplate reader (BMG Labtech, Cary, NC, USA).

### 2.9. Statistical Analysis

Statistical analysis was carried out using Minitab 17 software (Minitab Inc., State College, PA, USA) [16]. Statistically significant results were reported based on a one way analysis of variance (ANOVA) test followed by post hoc tests (Tukey’s/Fishers/Dunnett’s) [17,18]. All values are expressed as percentage of target control with mean ± standard error (SE).

## 3. Results

### 3.1. mAb Induced Effect on in Cell Viability

**Figure 2:**
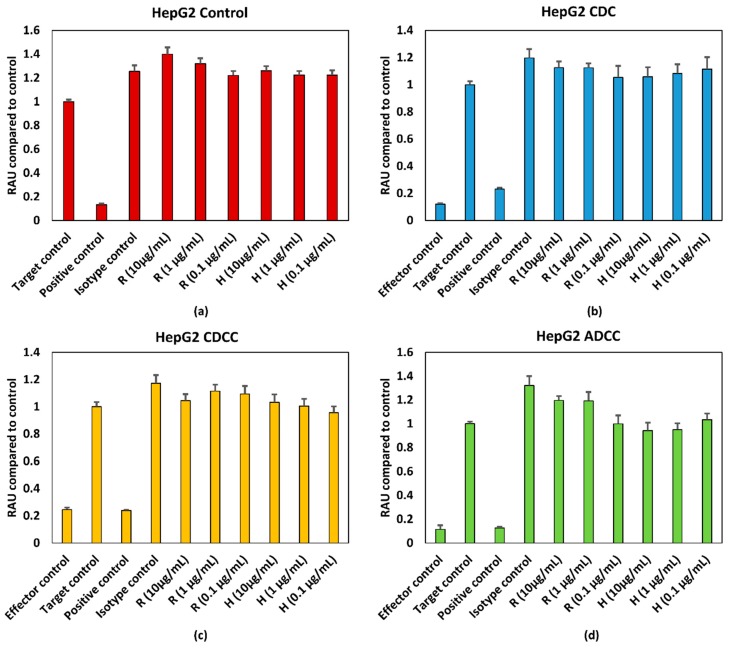
(**a**) Control conditions without effector cells/serum (**b**) CDC (**c**) CDCC, and (**d**) ADCC assay results of rituximab and Trastuzumab based on the WST-1 assay. Results represent pooled responses from four donors (*n* = 4). All values are expressed as Relative Absorbance Units (RAU) of control (mean ± SE). R: Rituximab and H: Trastuzumab. Positive control is 5% (*v*/*v*) of absolute ethanol. HepG2: hepatocarcinoma cell line.

HepG2 cells were treated with varying concentrations of rituximab and trastuzumab. Cell viability was expressed as percentage of Target control which are wells containing only HepG2 cells. Effector cells/serum blank refer to those wells which contain only PBMCs for ADCC, serum for CDC and PBMCs + serum for CDCC assays to measure background absorbance for the effector cells and/or serum for the respective endpoint. Neither a concentration dependent effect on cell viability nor an effector/serum dependent response were observed for either of the tested mAbs as shown in Figure 2, 2.5% (*v*/*v*) of ethanol (final concentration) was used as the positive control. Figure 2b–d represents the pooled responses from four donors for CDC, CDCC and ADCC tests. 

### 3.2. mAb Induced Effect in ATP Levels

**Figure 3:**
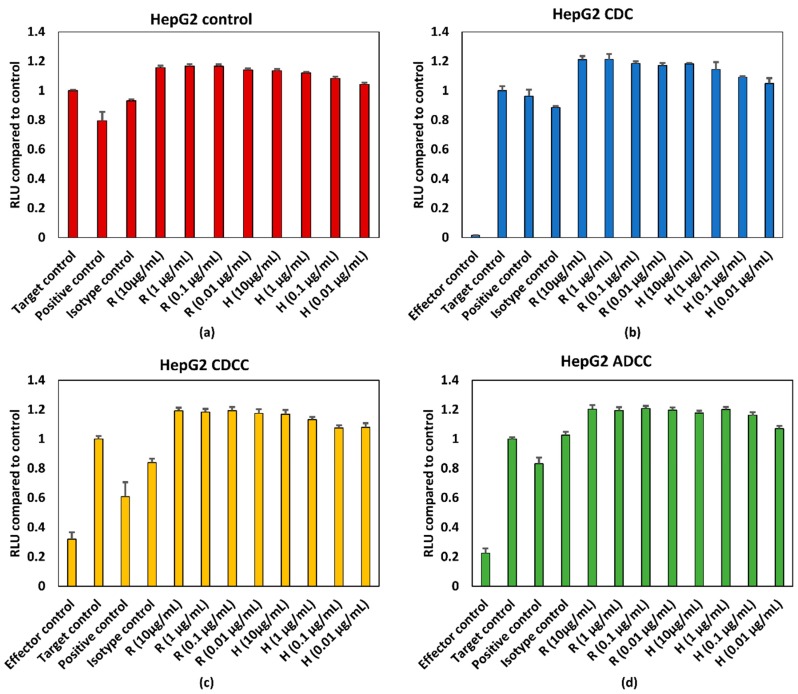
(**a**) Control conditions without effector cells/serum, (**b**) CDC, (**c**) CDCC, and (**d**) ADCC assay results of rituximab and trastuzumab based on the ATP content compared to control in HepG2 cells exposed to mAbs. Results represent pooled responses from four donors (*n* = 4). All values expressed as relative luminescence units (RLU) compared to control (mean ± SE). R: Rituximab and H: Trastuzumab. Positive control is 5% (*v*/*v*) of absolute ethanol.

**Figure 4:**
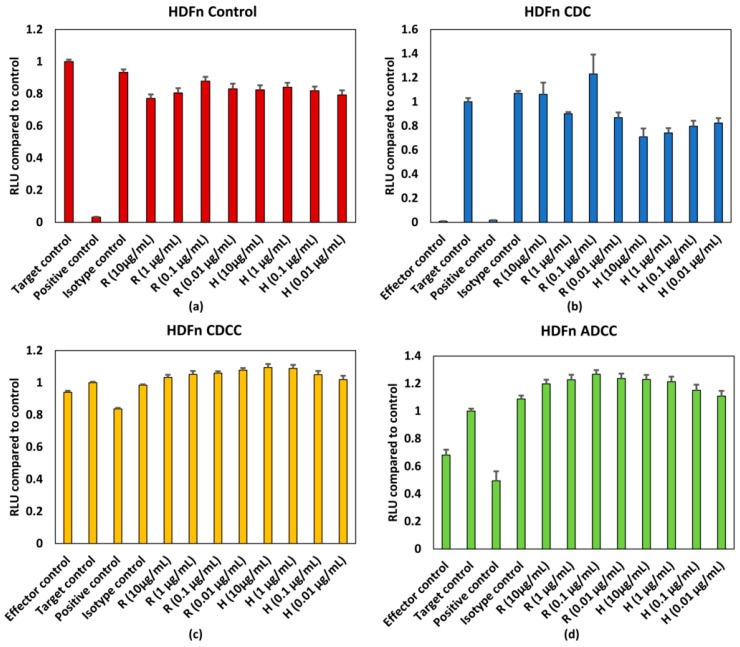
(**a**) Control conditions without effector cells/serum, (**b**) CDC, (**c**) CDCC, and (**d**) ADCC assay results of rituximab and Trastuzumab based on the ATP content compared to control in HDFn cells exposed to mAbs. Results represent pooled responses from four donors (*n* = 4). All values expressed as relative luminescence units (RLU) compared to control (mean ± SE). R: Rituximab and H: Trastuzumab. Positive control is 5% (*v*/*v*) of absolute ethanol.

HepG2 and HDFn cells were treated with varying concentrations of rituximab and trastuzumab. ATP content was expressed as percentage of target control which are wells containing only HepG2 cells or HDFn cells. A final concentration of 5% (*v*/*v*) ethanol was used as the positive control. Effector cells/serum blank refer to those wells which contain only PBMCs for ADCC, serum for CDC and PBMCs + serum for CDCC assays to measure background absorbance for the effector cells and/or serum for the respective endpoint. Figure 3 and Figure 4 represent pooled responses from four donors for ADCC, CDC, and CDCC tests. Neither a concentration dependent effect on ATP content nor an effector/serum dependent response were observed for either of the tested mAbs on HepG2 cells (Figure 3).

The HDFN cells seem to be slightly more sensitive to the responses evoked by mAbs when compared to HepG2 cells. Figure 4a depicts the response elicited by mAbs without the influence of PBMCs and/or serum. The response is generally lower than the target control, which contains only HDFn cells. When assessing responses resulting from CDC (Figure 4b), all concentrations of trastuzumab tested have lower responses when compared to the control. A similar trend can be observed for rituximab, however the variation is higher when compared to trastuzumab (Figure 4b). Neither a concentration dependent effect on ATP content nor an effector/serum dependent response were observed for either of the tested mAbs resulting from ADCC and CDCC (Figure 4c,d). HDFn cell seem to be more sensitive to responses elicited by mAbs. 

### 3.3. Effect of Donor Variability and Intrinsic Variation

**Figure 5:**
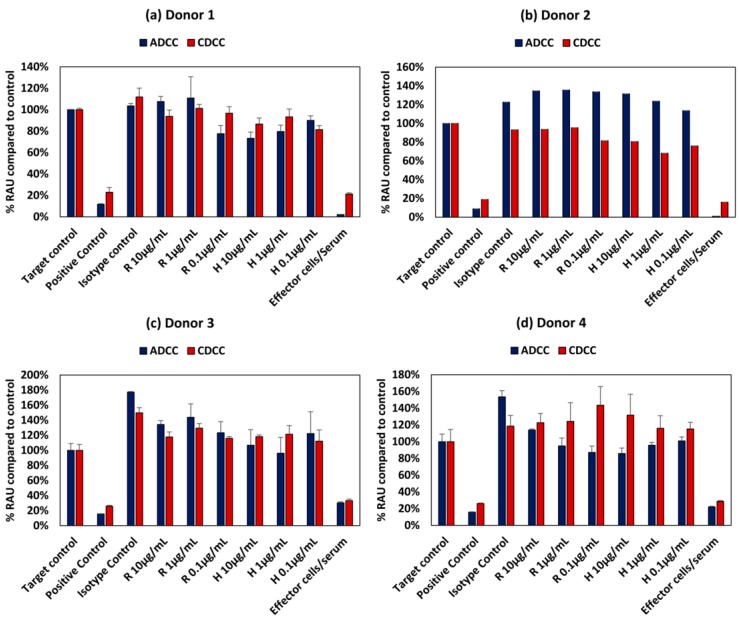
Intrinsic variation in responses owing to donor variability in ADCC and CDCC assay results of rituximab and Trastuzumab based on the ATP content compared to control in HepG2 cells exposed to mAbs for (**a**) Donor 1, (**b**) Donor 2, (**c**) Donor 3 and (**d**) Donor 4 All values expressed as relative Absorbance Units (RAU) compared to control (mean ± SE). R: Rituximab and H: Trastuzumab. Positive control is 5% (*v*/*v*) of absolute ethanol.

The PBMCs used in the assay were obtained from four different donors and the corresponding responses were varied and non-specific both in terms of the mAb used as well as dose. Figure 5 shows the intrinsic variability in the ADCC and CDC assays owing to donor variability. This variability could be due the specificity of the immune response evoked by each individual which depends on many factors such as genetic make-up and environmental exposure. This intrinsic variation in the assay could potentially confound the outcome of any adverse effect elicited by mAbs.

## 4. Discussion

The in vitro systems selected in this study were based on the two main adverse effects associated with mAb based therapeutics: hepatotoxicity and dermal toxicity. Rituximab and trastuzumab elicit an immune mediated reaction to neutralize tumour cells via ADCC, CDC, and/or CDCC [5,15]. The traditional toxicity tests used here are routinely used for assessing safety and toxicity of compounds in multitier toxicological assessment studies [6]. The objective of the assay used here was to observe any adverse effects of mAbs on HepG2 and HDFn cells upon exposure to naïve PBMCs i.e., detection of any off-target toxicity elicited by mAbs. Both Rituximab and trastuzumab have shown hepatobiliary and skin/infusion related adverse effects in clinical trials [9,10]. 

However, as seen from the results shown in Figure 2 and Figure 3, no dose dependent effect on cell viability or ATP levels were observed for either of the mAbs for HepG2 cells. The antigens for rituximab and trastuzumab are CD20 and HER2, respectively. As the potential off target effects were investigated, both cell lines were chosen such that they do not possess these antigens as surface markers. As HepG2 and HDFn cells do not express the antigen for either rituximab or trastuzumab ADCC and CDCC modes of decrease in cell viability were not observed owing to lack of direct cross target binding associated toxicity. While rituximab has shown to elicit higher CDC mediated responses, the CDC mediated effect of trastuzumab is comparatively lower [15]. This has shown to be due to the influence of membrane-bound complement regulatory proteins such as CD46, CD55, and CD59 which are overexpressed in tumour cells [19]. CD46 is indeed overexpressed in HepG2 cells and this could be an additional reason why CDC mediated effect was not observed in HepG2 as compared to HDFn cells [20]. For HDFn cells a decrease in ATP compared to control was observed for all concentrations of trastuzumab tested and a similar trend was observed for rituximab but with higher variability, for CDC mediated response (Figure 4b).

Variability in donor responses will be a confounding factor affecting the potential to detect any off target adverse effects, as intra donor variability is quite high owing to the specificity of immune responses elicited (Figure 5). The clinical immunogenicity associated with rituximab and Trastuzumab are between 1–23% and 1%, respectively, and this variation is reflective of clinical trials. [21].

Exposure time is another possible reason for the lack of response for these assays. Immune specific reaction could take 5–7 days to develop and this could lead to depletion in the target cell number and hence wouldn’t be feasible in this context without a continuous culture in place. This has been shown in T cell proliferation assays for monoclonal antibodies wherein the early phase effects were identified at 20 h and late phase effects at 7 days [21]. However, this requires additional measures in place for continuous maintenance of the PBMCs to maintain them at least a minimum 90% viability. In the case of traditional toxicity tests used to assess off target effects, a continuous maintenance of PBMCs would confound the ability of the test to detect any decrease in cell viability. The percentage viability of PBMCs compared to control at the end of the 72 h testing period was below 40% as seen from Figure 2c,d and Figure 3c,d for HepG2 based testing and this distorts the effector target ratio essential for achieving a response. The percentage viability of PBMCs compared to control was around 70% for ADCC and around 85% for CDCC as seen in Figure 4c,d for HDFn cells.

The hallmarks of state of the art non-clinical safety testing tools that would facilitate the accelerated growth of the biopharmaceutical market would be: high throughput; rapid and cost effective; highly reproducible and allow for early stage screening. They would also provide an alternative to animal testing considering the various drawbacks of in vivo systems as seen in the case of TGN1412 [22]. The evolution of simple 2D systems to complex in vitro systems such as 3D spheroidal co-cultures, organs on chips as well as whole blood systems are platforms that are better representations of immune responses elicited in humans [23]. Receptor binding studies are also considered to be indicative of biological activity of mAbs as binding to different FcγR receptors elicit different effector functions [24]. These studies can either be cell based or conjugated beads based such as αscreen™ technology [25,26]. Immunogenicity testing of mAb based therapeutics using T cell proliferation and cytokine assay have been reported previously for rituximab and trastuzumab [21]. Hypersensitivity reactions have been assessed using Skimune™, a non-artificial human skin explants based assay for safety and efficacy assessment of novel compounds and drugs, developed by Alcyomics Ltd., Newcastle upon Tyne, UK [27]. Immunoinformatics is another promising area which allows for assessing the presence of potential T cell and B cell epitopes that could lead to formation of anti-idiotype antibodies as well as an aggravated immune response [28,29]. These advancements may contribute to enhanced non-clinical safety testing strategies for mAb developability.

To summarize, this study looked at the applicability of traditional in vitro toxicity tests to assess potential off target hepatotoxicity and dermal toxicity of mAbs using HepG2 and HDFn cell based assays. Though these assays are routinely using for assessing toxicity of compounds, they deemed to be unsuitable in this case due to several factors and thus, no apparent dose or mAbs specific cytotoxicity or decrease in ATP levels were observed. Therefore, novel assays would be more suitable for detecting potential immune related adverse effect elicited by mAbs. 

## Appendix A

**Table A1:** Details of the final mAb concentration and associated dilutions.

Working Concentrations	Concentration in Well (µg/mL)	Volume of mAb (µL)	Volume of DMEM (µL)	Source	Final Volume in Well (µL)	Volume Added/Well (µL)
40	10	48	1152	1 mg/mL	200	50
4	1	120	1080	40	200	50
10.4	0.1	120	1080	4	200	50

Working concentration = Concentration in well × dilution factor (4 as total volume in cell is 200 µL) Rituximab stock is 100 mg/mL; Working stock of 10 mg/mL: 100 µL in 900 µL of PBS
